# A Case-Control Study of the Association between Vitamin D Levels and Gastric Incomplete Intestinal Metaplasia

**DOI:** 10.3390/nu10050629

**Published:** 2018-05-16

**Authors:** Kevin Singh, Soren Gandhi, Raffat Batool

**Affiliations:** 1 Department of Internal Medicine, New York University School of Medicine, Woodhull Medical and Mental Health Center, Brooklyn, New York, NY 11206, USA; 2 Department of Gastroenterology and Hepatology, New York University School of Medicine, Woodhull Medical and Mental Health Center, Brooklyn, New York, NY 11206, USA; soren.gandhi@nychhc.org (S.G.); raffat.batool@nychhc.org (R.B.); 3 Department of Gastroenterology and Hepatology, New York Medical College, Metropolitan Hospital Center, New York, NY 10029, USA

**Keywords:** gastric adenocarcinoma, intestinal metaplasia, vitamin D deficiency

## Abstract

**Aim**: Low circulating vitamin D levels are associated with gastric adenocarcinoma, but whether vitamin D levels are associated with premalignant gastric mucosal changes is unknown. Here, we determined associations between vitamin D levels and gastric incomplete intestinal metaplasia, a known gastric adenocarcinoma risk factor. **Methods**: This was a retrospective, unmatched, case-control study comparing serum 25-hydroxyvitamin D levels among subjects with gastric incomplete intestinal metaplasia (cases; *n* = 103) and those without gastric incomplete intestinal metaplasia (controls; *n* = 216). The 25-hydroxyvitamin D levels were categorized as normal (30–100 ng/dL), vitamin D insufficiency (VDi; 20–29 ng/dL), and vitamin D deficiency (VDd; <20 ng/dL). Using multivariable logistic regression, odds ratios (ORs) were calculated and adjusted to age, gender, ethnicity, body mass index, history of hypertension or diabetes mellitus, and timing of vitamin D collection to assess associations between 25-hydroxyvitamin D levels and gastric incomplete intestinal metaplasia. Results: A majority of case subjects were male, Hispanic, and did not have hypertension or diabetes mellitus. The average serum 25-hydroxyvitamin D level was significantly lower in the intestinal metaplasia group than the control group (19.7 ng/dL vs. 34.7 ng/dL; *p* < 0.001). Hypovitaminosis D was more common in subjects with incomplete intestinal metaplasia in a multivariable regression model (OR 54.1, 95% CI 21.8–134.3; *p* < 0.001). VDd (OR 129.0, 95% CI 43.7–381.2; *p* < 0.001) and VDi (OR 31.0, 95% CI 11.9–80.3; *p* < 0.001) were more common in patients with incomplete intestinal metaplasia than healthy subjects, with VDd slightly more prevalent than VDi (OR 4.0, 95% CI 1.7–9.6; *p* < 0.001). **Conclusions**: Vitamin D deficiency and insufficiency are more common in patients with gastric incomplete intestinal metaplasia than healthy subjects and may play a role in the development of premalignant phenotypes related to gastric adenocarcinoma.

## 1. Introduction

Gastric adenocarcinoma is the fourth most prevalent cancer worldwide. Patients with gastric adenocarcinoma are often asymptomatic prior to diagnosis so present with late-stage disease and have a poor prognosis, the five-year survival rate for advanced disease being less than 30% [1,2]. Chronic exposure to carcinogens, primarily *Helicobacter pylori* infection but also to a lesser degree, tobacco and alcohol consumption, results in gastric mucosal injury and sequential pathological changes that ultimately result in gastric adenocarcinoma in the absence of intervention [3,4]. This carcinogenetic process can be described according to the “Correa cascade”, a model that describes the stepwise histopathological progression from chronic atrophic gastritis to intestinal metaplasia, gastric dysplasia, and eventually adenocarcinoma [5].

Intestinal metaplasia is the most frequently observed precancerous change in the gastric mucosa. Although the absolute risk of progression from gastric intestinal metaplasia to gastric adenocarcinoma is relatively low in areas of low prevalence for gastric adenocarcinoma like North America and Europe, the prevalence is higher in other regions of the world including Southeast Asia. Intestinal metaplasia is characterized by the replacement of the gastric epithelium with intestinal-like epithelium, both of which are columnar in morphology. There are two subtypes of intestinal metaplasia, complete and incomplete, the former resembling the small intestine (mucin-rich, intestinal-like columnar epithelium with goblet cells, a brush border, and eosinophilic enterocytes) and the latter resembling the large intestine, with distinguishing features including re-expression of gastric mucins and less digestive enzymes [5]. Incomplete intestinal metaplasia, often considered a mild form of gastric dysplasia [6,7], carries a 4- to 11-fold increase in risk for the development of gastric adenocarcinoma compared with complete intestinal metaplasia.

However, enrollment in endoscopic surveillance programs to detect gastric dysplasia and early gastric adenocarcinoma is controversial, especially in low-prevalence areas including in the United States. The American Society of Gastrointestinal Endoscopy does not recommend surveillance endoscopy for patients with gastric intestinal metaplasia unless the patient is otherwise considered high risk due to a family history or ethnic predisposition. American Journal of Gastroenterology guidelines recommend surveillance endoscopy with biopsy mapping of the gastric mucosa or serum pepsinogen levels one year after the index endoscopy, with repeat surveillance endoscopy three years later if there is extensive intestinal metaplasia, atrophy, or incomplete intestinal metaplasia. The European Society of Gastrointestinal Endoscopy guidelines recommend surveillance endoscopy in patients with gastric intestinal metaplasia every three years [7]. Regardless, knowledge and prevention of risk factors [3,4,5] for intestinal metaplasia are crucial, because intestinal metaplasia rarely spontaneously regresses and patients that develop more advanced phenotypes such as gastric dysplasia or adenocarcinoma require curative but invasive procedures such as endoscopic mucosal resection, endoscopic submucosal dissection, or gastrectomy, and outcomes are poor with advanced disease. 

Vitamin D is a fat-soluble vitamin that, in its biologically active form of 1,25-dihydroxyvitamin D, has a defined role in bone and calcium metabolism. Due to its known antineoplastic and antioxidant properties, several studies have explored the association between vitamin D levels and cancer progression. Indeed, low vitamin D levels are associated with an increased risk of several malignancies including breast, prostate, colorectal, and pancreatic cancers [8,9,10,11,12], and vitamin D supplementation has been used for cancer prophylaxis [13]. There is also mounting evidence that low vitamin D levels are associated with an increased risk of gastric adenocarcinoma and may represent a poor prognostic factor [14]. However, to our knowledge, the relationship between vitamin D levels and the presence of gastric cancer precursor lesions such as gastric intestinal metaplasia has yet to be studied. Therefore, here we sought to determine if vitamin D levels are associated with gastric incomplete intestinal metaplasia.

## 2. Materials and Methods

### 2.1. Subjects

**Figure 1:**
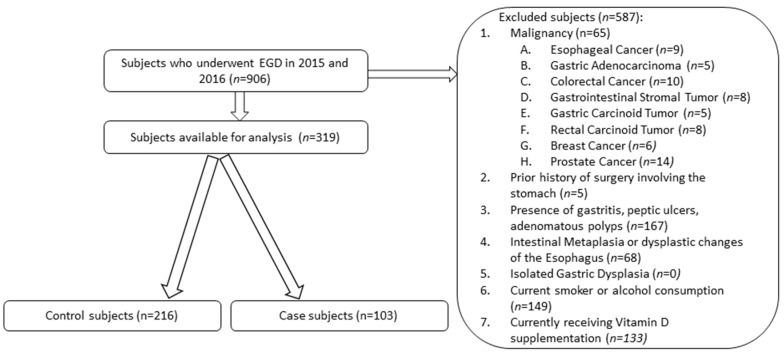
Flowchart describing the inclusion and exclusion criteria applied to the dataset.

Nine hundred and six subjects were recruited after undergoing esophagogastroduodenoscopy (EGD) during 2015 and 2016. All subjects were aged 18 years or older, had undergone EGD, and had documented serum 25-hydroxyvitamin D levels from population-based screening from their primary care provider two months prior to EGD and repletion. The 25-hydroxyvitamin D was selected as the biomarker of vitamin D levels since it is the predominant form of systemic circulating vitamin D and is the best indicator of overall vitamin D stores [15]. Subjects were excluded if they were younger than 18 years old, did not undergo EGD, did not have a documented serum 25-hydroxyvitamin D level prior to EGD, consumed alcohol or smoked cigarettes, had a prior surgical procedure involving the stomach, or had been diagnosed with gastric ulcers, adenomatous polyps, intestinal metaplasia or dysplasia of the esophagus, dysplasia of the stomach, or a concomitant malignancy (Figure 1). To ensure that patients did not previously smoke or consume alcohol at any point, multiple visits logged in the electronic medical record were reviewed to confirm their status for possible exclusion.

All patients were recruited from the gastroenterology practice at Woodhull Medical and Mental Health Center, an academically affiliated hospital of New York University School of Medicine located in Brooklyn, NY, USA. This hospital serves a diverse ethnic population with a predominance of Hispanics, African Americans, and, to a lesser degree, Asian Americans and Caucasians. 

### 2.2. Design

This was a single-center, retrospective, unmatched, case-control study of subjects who had undergone EGD and gastric biopsy for evaluation of dyspepsia. Based on the presence or absence of gastric incomplete intestinal metaplasia after gastric biopsy, patients were designated as either a control (no gastric intestinal metaplasia) or case (gastric intestinal metaplasia) subject. Serum 25-hydroxyvitamin D levels were classified according to definitions established by the Endocrine Society: 30–100 ng/dL was considered normal, with insufficient 25-hydroxyvitamin D levels or hypovitaminosis D subgrouped into two: vitamin D insufficiency (VDi; 20–29 ng/dL) and vitamin D deficiency (VDd; <20 ng/dL) [12,15]. Two categories were used to assess timing of vitamin D collection: fall-winter representing times of less daylight exposure (fall months included September, October, and November, and winter months included December, January, and February) and spring-summer, representative of months with more daylight exposure (spring months included March, April, and May, and summer months included June, July, and August). These categories were used because of the retrospective design of this study limiting the ability to reliably measure daylight exposure among different subjects, in addition to taking into account the two-month interval that was needed to collect vitamin D levels before EGD. Body mass index (BMI) was categorized according to the World Health Organization classification: underweight (<18.5 kg/m^2^), normal (18.5–24.9 kg/m^2^), overweight (25–29.9 kg/m^2^), and obesity class I (30–34.9), class II (35–39.9), and class III (≥40).

The mean and standard deviation (SD) of the case and control subjects’ ages, BMI, and 25-hydroxyvitamin D levels were calculated. Multivariable logistic regression was performed using IBM SPSS for Windows (version 23; IBM SPSS Inc., Armonk, NY, USA) to calculate odds ratios (ORs) to assess the association between 25-hydroxyvitamin D levels and gastric incomplete intestinal metaplasia. ORs were adjusted to confounders including age, gender, ethnicity, BMI, history of diabetes mellitus, hypertension, and timing of vitamin D collection, while including the three levels of vitamin D status as co-variates. To evaluate differences between cases and controls, continuous variables such as age, BMI, and average serum 25-hydroxyvitamin D levels were assessed using a 2-tailed paired *t*-test, and chi-squared analysis was used to test categorical variables such as gender, ethnicity, BMI class, history of diabetes mellitus or hypertension, and timing of vitamin D collection. A *p*-value less than 0.05 was considered statistically significant.

Because this study was retrospective by design, the Biomedical Research Alliance of New York (BRANY) exempted the study from Institutional Review Board (IRB) review since it met the standards set forth by the Declaration of Helsinki. Also, requirements for informed consent from subjects were waived by BRANY because of the retrospective design and complete anonymity of subjects.

## 3. Results

### 3.1. Demographic Information

Of the 906 recruited subjects, 319 subjects (approximately 35%) met the inclusion criteria (Figure 1). Age, gender, ethnicity, BMI, were similar among case and control subjects; case subjects were on average 59.4 years old, 50.5% were male, 68% were Hispanic, 96.1% did not have hypertension, and 87.4% of patients did not have diabetes mellitus. Among the case subjects, the 25-hydroxyvitamin D levels were collected predominantly during the spring and summer (75.7%), seasons with more daylight (Table 1). The control subjects were on average 56.9 years old, Hispanic (64.8%) and consisted of more females (67.5%). More control subjects had hypertension (18.1% vs. 3.9%) and had vitamin D collections during the fall and winter seasons (38.4% vs. 24.3%) than the case subjects.

### 3.2. Analysis of Vitamin D Status in Intestinal Metaplasia

The majority of control subjects had normal serum vitamin D levels (52.0%; Table 2). Case subjects with gastric incomplete intestinal metaplasia had a lower average serum 25-hydroxyvitamin D level (19.7 ng/dL) than control subjects 34.7 ng/dL (*p* < 0.001; Table 3).

Multivariable logistic regression was performed to assess the impact of confounders such as age, ethnicity, gender, BMI, diabetes mellitus, hypertension, and timing of vitamin D collection (Table 4). Hypovitaminosis D was much more common in subjects with gastric incomplete intestinal metaplasia (OR 54.1, 95% CI 21.8–134.3, *p* < 0.001; Table 2 and Table 4) compared to controls; gender was also statistically significant in this model (*p*-value 0.01; Table 4). Of the patients with gastric incomplete intestinal metaplasia and hypovitaminosis D, VDd (OR 129.0, 95% CI 43.7–381.2, *p* < 0.001) and VDi (OR 31.0, 95% CI 11.9–80.3, *p* < 0.001) were more prevalent than normal serum 25-hydroxyvitamin levels in patients with gastric incomplete intestinal metaplasia, with VDd slightly more prevalent than VDi (4.0, 95% CI 1.7–9.6, *p* < 0.001) in this group (Table 2).

In univariate analyses, among subjects with gastric incomplete intestinal metaplasia, average 25-hydroxyvitamin D levels were generally significantly lower in comparison with the control group and were generally similar even when divided by subgroup (Table 3). Serum 25-hydroxyvitamin D levels were even lower in subjects with intestinal metaplasia without hypertension than hypertensive patients. However, an association between low vitamin D levels and intestinal metaplasia could not be made in diabetic subjects. Asians (*n* = 4) with incomplete intestinal metaplasia had the lowest average 25-hydroxyvitamin D levels followed by Hispanic, Caucasian, and African American patients.

## 4. Discussion

Gastric adenocarcinoma is an aggressive malignancy with a poor prognosis and limited therapeutic options, especially at later stages. Preventative strategies in at-risk patients involve avoiding or treating known carcinogenic risk factors. One potential modifiable risk factor is vitamin D, an antioxidant and anti-cancer agent in vitro [16,17,18,19,20,21,22,23,24,25]. Vitamin D arrests carcinogenesis via upstream modulation of gene transcription by the vitamin D receptor (VDR). After fat-soluble 1,25-dihydroxyvitamin D traverses cell membranes, it binds to cytoplasmic VDR [25] followed by vitamin D-VDR complex binding to the retinoid X receptor (RXR). This ligand-complex translocates into the nucleus to bind chromatin and activate transcription via histone acetyl transferase and ATP-dependent chromatin remodeling to provide transcription factor access to vitamin D response elements (VDREs) at gene promoters. The downstream functional effects of this pathway include induction of apoptosis. In further support of the pathogenetic role of vitamin D in gastric cancer, subjects with premalignant and malignant histopathological changes were found to have decreased VDR expression [24]. Further, CD24 mediates gastric adenocarcinoma cell survival and invasion by activating STAT3 signaling and regulating extracellular matrix protein and VDR expression [20]. Taken together, these data suggest that reduction of VDR expression may facilitate evasion of apoptosis and affect the cell cycle to promote carcinogenesis. 

Since retrospective studies have demonstrated an association between hypovitaminosis D (vitamin D insufficiency and deficiency) and gastric adenocarcinoma, low vitamin D levels may also be associated with other steps in the carcinogenetic pathway [10,11,12]. However, no study has yet assessed associations between hypovitaminosis D and pre-malignant histopathological changes in the gastric mucosa. Here, we fill this knowledge gap by assessing the association between circulating vitamin D levels and incomplete intestinal metaplasia using a retrospective case-control study design. VDd and VDi were more common in patients with incomplete intestinal metaplasia than healthy subjects and, overall, more subjects with incomplete intestinal metaplasia had VDd than VDi. The average serum 25-hydroxyvitamin D level was significantly lower in subjects with incomplete intestinal metaplasia than in healthy subjects, supporting a hypothesis that low serum vitamin D levels may play a role in gastric carcinogenesis as early as the intestinal metaplasia stage of development. 

In a subgroup analysis, patients with incomplete intestinal metaplasia had lower overall 25-hydroxyvitamin D levels than patients without intestinal metaplasia in all categories except for in diabetics and included age, gender, ethnicity, BMI, or hypertension. These findings were also relevant when considering timing of vitamin D collection. Even though collections were predominantly in the spring and summer, case subjects had lower serum 25-hydroxyvitamin D levels than control subjects in both groups. In addition, hypovitaminosis D was most common in Asians with incomplete intestinal metaplasia, but firm conclusions about ethnic differences in serum 25-hydroxyvitamin D levels in patients with incomplete intestinal metaplasia are difficult to make due to the relatively small sample size and very few included Asian and Caucasian subjects. Furthermore, although vitamin D levels may be artificially low in patients with excess adipose tissue, such as in obese subjects, the vitamin D levels were generally similar in both case and control subjects across BMI classes, so this effect was not observed in our study. 

Our study has several limitations. The sample size was relatively small, so the confidence intervals were broad, and validation in larger studies will be needed to confirm our observations. Due to the imbalanced ethnic demography, our studies may not be generalizable to all ethnic groups, which is particularly important given that gastric adenocarcinoma is most prevalent among Asian populations [12]. Also, though *H. pylori* infection is the primary mediator of intestinal metaplasia, there are other etiological risk factors including even moderate alcohol consumption and cigarette smoking, and these patient populations were excluded so we cannot generalize our results to these populations [26,27]. The prevalence of complete intestinal metaplasia varies, with an overall prevalence of 19%, accounting for 8.2–86% of patients with intestinal metaplasia in some studies [28,29,30]. In our population, there was not only a low prevalence of isolated complete intestinal metaplasia but also gastric dysplasia in the absence of an exclusion criterion; as a result, we could not make conclusions regarding the association between vitamin D and these other premalignant changes. Lastly, because this study was retrospective, causality cannot be proven, and prospective studies will be needed to confirm these initial findings and determine a causative role for hypovitaminosis D and gastric incomplete intestinal metaplasia.

In conclusion, low serum vitamin D levels may play a role in the development of incomplete intestinal metaplasia in the gastric mucosa. Future prospective studies are needed to definitively establish if low vitamin D levels contribute to intestinal metaplasia and, if causation is proven, study of the mechanism by which hypovitaminosis D mediates carcinogenesis at the stage of intestinal metaplasia and beyond is warranted. Given these findings, chemoprophylaxis with vitamin D may be a relatively simple intervention to prevent the development and progression of gastric adenocarcinoma and should be pursued in interventional trials, not least because other antioxidants such as ascorbic acid and β-carotene have been shown to be effective for chemoprophylaxis for gastric adenocarcinoma in patients with intestinal metaplasia [31].

## Figures and Tables

**Table 1:** Demographic information of case and control subjects.

	Intestinal Metaplasia (Cases)	No Intestinal Metaplasia (Controls)	*p*-Value
Number of subjects (*N*)	103	216	
Age (years)	59.4 ± 11.6	56.9 ± 13.6	NS ^1^
<60 years old	49.5% (51)	61.1% (132)	
>60 years old	50.5% (52)	38.9% (84)	
Gender			0.01
Male	50.5% (52)	32.4% (70)	
Female	49.5% (51)	67.5% (146)	
Ethnicity			NS
Hispanic	68% (70)	64.8% (140)	
African American	23.3% (24)	19.0% (41)	
Caucasian	4.9% (5)	13.0% (28)	
Asian	3.9% (4)	3.2% (7)	
Body mass index (BMI; kg/m^2^)			NS
Underweight	3.9% (4)	1.9% (4)	
Normal	24.3% (25)	24.1% (52)	
Overweight	35.9% (37)	34.7% (75)	
Obese (total)	35.9% (37)	39.3% (85)	
Obesity Class I	24.3% (25)	20.4% (44)	
Obesity Class II	10.7% (11)	12% (26)	
Obesity Class III	1% (1)	6.9% (15)	
History of Diabetes Mellitus			NS
Without Diabetes Mellitus	87.4% (90)	91.7% (198)	
With Diabetes Mellitus	12.6% (13)	8.3% (18)	
History of hypertension			<0.001
Without Hypertension	96.1% (99)	81.9% (177)	
With hypertension	3.9% (4)	18.1% (39)	
Season of vitamin D measurement			0.01
Fall-Winter	24.3% (25)	38.4% (86)	
Spring-Summer	75.7% (78)	61.6% (133)	

^1^ Abbreviations: NS: Not Significant.

**Table 2:** Comparison of vitamin D states among patients with and without intestinal metaplasia.

Vitamin D Status	Intestinal Metaplasia (Cases)	No Intestinal Metaplasia (Controls)
Normal vitamin D level (30–100 ng/dL)	2.2% (7)	52.0% (166)
Hypovitaminosis D (<30 ng/dL)	97.8% (96)	48.0% (50)
Vitamin D insufficiency (VDi) (20–30 ng/dL)	43.8% (42)	78% (39)
Vitamin D deficiency (VDd) (<20 ng/dL)	56.3% (54)	22% (11)
	Odds ratio (95% CI)	*p*-value
Normal vitamin D levels		
versus		
VDd and VDi	54.1 (21.8–134.3)	<0.001
VDd	129.0 (43.7–381.2)	<0.001
VDi	31.0 (11.9–80.3)	<0.001
VDd versus Vdi	4.0 (1.7–9.6)	<0.001

Abbreviations: VDi: vitamin D insufficiency (20–29 ng/dL); VDd: vitamin D deficiency (<20 ng/dL).

**Table 3:** Average vitamin D levels among subjects with and without gastric incomplete intestinal metaplasia.

	Intestinal Metaplasia (Cases)	No Intestinal Metaplasia (Controls)	*p*-Value
Overall vitamin D level (ng/dL)	19.7 ± 6.3	34.7 ± 10.0	<0.001
Variables			
Age			
<60 years old	19.8 ± 6.5	35.2 ± 11.2	<0.001
>60 years old	19.7 ± 6.3	34.0 ± 8.0	<0.001
Gender			
Male	20.4 ± 7.0	34.4 ± 10.4	<0.001
Female	19.9 ± 6.6	34.8 ± 9.9	<0.001
BMI class			
Underweight	24.3 ± 2.6	42.0 ± 22.2	0.25
Normal	19.4 ± 6.5	34.5 ± 9.6	<0.001
Overweight	19.7 ± 7.9	35.7 ± 10.8	<0.001
Obese (pooled)	20.4 ± 6.1	33.5 ± 8.8	<0.001
Obesity class I	21.1 ± 6.1	33.9 ± 8.9	<0.001
Obesity class II	19.6 ± 6.4	33.1 ± 9.5	0.03
Obesity class III	20.5 *	33.2 ± 7.4	N/A *
Ethnicity			
Hispanic	19.3 ± 6.1	34.4 ± 10.8	<0.001
African American	21.5 ± 6.6	34.6 ± 8.1	<0.001
Caucasian	20.9 ± 7.8	36.2 ± 9.5	<0.001
Asian	14.05 ± 3.8	35.2 ± 4.6	<0.001
Blood pressure			
Normotensive patients	19.6 ± 6.8	34.8 ± 10.1	<0.001
Hypertensive patients	23.6 ± 6.3	34.4 ± 10.0	<0.001
History of diabetes mellitus			
Without diabetes mellitus	19.5 ± 6.3	34.4 ± 9.1	<0.001
With diabetes mellitus	25.7 ± 5.0	38.4 ± 17.33	NS
Timing of Vitamin D Collection			
Fall-Winter	22.0 ± 7.3	34.1 ± 9.9	<0.001
Spring-Summer	19.6 ± 6.6	35.0 ± 10.1	<0.001

* One subject with intestinal metaplasia with obesity class III, so a SD could not be calculated for the case subjects within this class and a *t*-test could not be performed.

**Table 4:** Multivariable logistic regression analysis including confounders of age, ethnicity, gender, BMI, history of hypertension or diabetes mellitus, and timing of vitamin D collection.

Variable	*B*	Standard Error	Wald Statistic	*p*-Value	OR, 95% CI
Hypovitaminosis D	4.0	0.46	73.9	<0.001	54.1 (21.8–134.3)
Age	0.004	0.01	0.10	0.76	1.00 (0.98–1.03)
Ethnicity	−19.24	2 × 10^4^	3.89	0.27	0.000
Gender	−1.00	0.37	7.03	0.008	0.37 (0.18–0.77)
BMI	−0.06	0.14	0.17	0.68	0.95 (0.72–1.23)
Diabetes mellitus	−1.30	0.84	2.42	0.12	1.40 (0.05–1.40)
Hypertension	0.22	0.51	0.18	0.67	1.24 (0.46–3.35)
Timing of vitamin D collection	0.69	0.38	3.23	0.07	1.99 (0.94–4.23)
Constant	−2.92	1.73	2.86	0.1	0.05
